# From Attraction to Repulsion to Attraction: Non-monotonic Temperature Dependence of Polymer-Mediated Interactions in Colloidal Dispersions

**DOI:** 10.1021/acsnanoscienceau.1c00011

**Published:** 2021-08-25

**Authors:** Sara Haddadi, Marie Skepö, Jan Forsman

**Affiliations:** Theoretical Chemistry, Lund University, P.O. Box 124, S-221 00 Lund, Sweden

**Keywords:** polystyrene particles, repulsive interactions, particle condensation

## Abstract

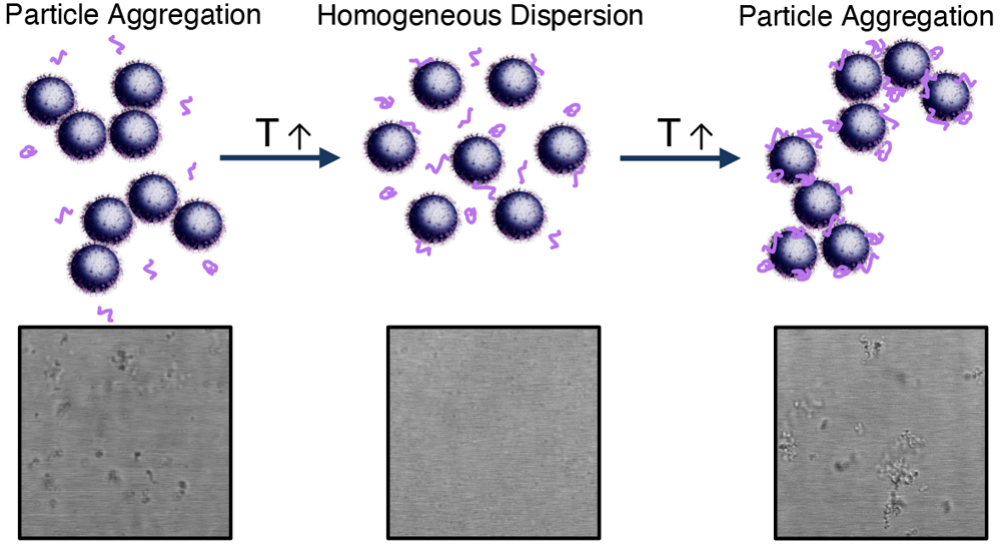

In this work, we have synthesized polystyrene particles that carry short end-grafted polyethylene glycol (PEG) chains. We then added dissolved 100 kDa PEG polymers and monitored potential flocculation by confocal microscopy. Qualitative predictions, based on previous theoretical developments in this field (Xie, F.; et al. *Soft Matter***2016**, *12*, 658), suggest a non-monotonic temperature response. These theories propose that the “free” (dissolved) polymers will mediate attractive depletion interactions at room temperature, with a concomitant clustering/flocculation at a sufficiently high polymer concentration. At high temperatures, where the solvent is poorer, this is predicted to be replaced by attractive bridging interactions, again resulting in particle condensation. Interestingly enough, our theoretical framework, based on classical density functional theory, predicts an intermediate temperature regime where the polymer-mediated interactions are *repulsive*! This obviously implies a homogeneous dispersion in this regime. These qualitative predictions have been experimentally tested and confirmed in this work, where flocs of particles start to form at room temperature for a high enough polymer dosage. At temperatures near 45 °C, the flocs redisperse, and we obtain a homogeneous sample. However, samples at about 75 °C will again display clusters and eventually phase separation. Using results from these studies, we have been able to fine-tune parameters of our coarse-grained theoretical model, resulting in predictions of temperature-dependent stability that display semiquantitative accuracy. A crucial aspect is that under “intermediate” conditions, where the polymers neither adsorb nor desorb at the particle surfaces, the polymer-mediated equilibrium interaction is *repulsive*.

## Introduction

Colloidal dispersions have been the subject of many studies, with focus ranging from stability and drug delivery, to self-assembly, patch formation, and so on.^1−5^ The stability of such dispersions can often be controlled by pH and temperature variation, or with additives, such as salt or polymers.^6−43^ One advantage in a system where polymers are used is their ability to mediate attractive, as well as repulsive, interactions between the particles, depending on parameters such as polymer length, solvent quality, or the presence of grafting bonds to the particles. This may facilitate a controlled stability.^10−15^ Crystallization of colloidal particles has also been investigated in many soft matter studies, and the coexistence between “gas” and ”liquid” phases can often be regulated by polymer properties.^16,17^ Previous work^18,19^ has established thermoresponsive aggregation, or cluster formation, in aqueous dispersions containing polystyrene (PS) particles, carrying a layer of grafted short polyethylene glycol (PEG) chains. A temperature increase from an ambient value, at which the dispersion remained sterically stabilized, caused a reduced solubility of the polymer grafts, which eventually resulted in gelation, aggregation, or cluster formation. An interesting result in the work by Shay et al.^19^ was that gelation occurred at temperatures far below the lower critical solution temperature (LCST) of an aqueous PEG solution.

The addition of non-adsorbing polymers to particle dispersions can also generate aggregation.^20−24^ In this case, the origin of the net attraction is an osmotic pressure difference as polymers are depleted from the configurationally restricted interparticle regions. Adsorbing polymers can also mediate attractive interactions, but in these cases, the underlying mechanism is instead attractive polymer bridges connecting the particles. The length scale of forces generated by polymer addition can be varied by the degree of polymerization, the concentration, and the polymer structure.^17^

In a recent publication, Feng et al.^22^ reported interesting re-entrant flocculation behavior of colloidal particles in an aqueous dispersion containing polymers. Several different polymer and particle combinations were investigated, but the ones displaying the re-entrant behavior had one common property: a solvent (water) that becomes poorer (for the polymer) as the temperature was increased. In those systems, a crystalline colloidal phase at low temperatures melts to a homogeneous fluid upon increasing the temperature. A further temperature increase, however, eventually gives rise to a flocculated phase. We have recently developed a model for such systems.^25^ In this model, monomers are assumed to be in either of two classes of states (rotational isomers), labeled **A** and **B**, where **B** is more solvophobic than **A**. On the other hand, the degeneracy of the **B** class exceeds that of **A**; that is, the population of solvophobic monomers increases with temperature. This can lead to an LCST.^26−28^ Adding solvophobic colloidal particles to such a solution generates a system that displays the same qualitative temperature response as was observed by Feng et al. That is, at low temperatures, type **A** monomers predominate and one observes depletion interactions, whereas polymer bridging dominates at higher temperatures due to the attraction between type **B** monomers and the colloidal surface. Interestingly, the intermediate temperature regime is characterized by a polymer-mediated potential of mean force (PMF) between colloidal particles, which is *repulsive*. This is a crucial and general theoretical observation, which was verified for systems for which one can obtain *exact* solutions (theta solvent conditions, with polymers modeled by connected point-like monomers). As surfaces are gradually modified from being nonadsorbing to adsorbing, there is an intermediate regime where polymer-mediated interactions indeed are repulsive. In other words, interactions between colloidal particles, mediated by neutral nongrafted polymers, generally follow the trend: attraction → repulsion → attraction as the surface affinity toward monomers proceeds from repulsive to attractive.^29^

Inspired by our recent studies,^18,29^ we here aim for a sort of theory–experiment loop. That is, armed with the theoretical progress described above, we can make qualitative predictions from which we design an experimental system. The measured temperature response of the latter will then permit the construction of a more targeted (semiquantitative) theoretical model by the adjustment of a few parameters. Specifically, we will perform measurements on PS particles carrying short (2 kDa) PEG chains grafted onto their surfaces. These are dispersed in an aqueous dispersion that also contains dissolved (“free”) PEG polymers, having a molecular weight of 100 kDa. The **A**/**B** state description of PEG monomer rotational isomers does seem to capture the essential physics underlying the presence of an LCST in aqueous solution. However, while the model is elegant, it is also somewhat complicated, and it might be desirable to simplify the model to one with fewer parameters, at the expense of a less detailed treatment of separate entropy/energy contributions. In this work, we have chosen to describe the effective degree of hydrophobicity of the PEG monomers, via the strength of a simple Lennard-Jones (L-J) potential, acting between all monomers. The L-J potential acting between the monomers will then be regarded as an interaction *free* energy (PMF) in this model. While we have not made attempts to establish quantitative relations between this L-J potential and a specific **A**/**B** model, the qualitative predictions by the latter do suggest more attractive L-J interactions at elevated temperatures even compared to thermal energy (*kT*). Using the simpler L-J description, we do sacrifice the mechanistic origin of the increased attraction (between monomers as well as between a monomer and a hydrophobic surface) at elevated temperatures.

Guided by the general theoretical predictions described above, we anticipate depletion-generated flocculation at room temperature, where water is a good solvent, at sufficiently high concentrations of dissolved (free) PEG. On the other hand, at high temperatures, PEG monomers become hydrophobic and thus attract. There is also an expected affinity between these monomers and the hydrophobic bare PS surface. Hence, bridging attraction should lead to flocculation at high temperatures. Finally, our theoretical considerations suggest that there is an intermediate temperature regime where dissolved PEG chains mediate repulsive interactions, which should facilitate a stable homogeneous dispersion.

Confocal laser scanning microscopy (CLSM) and dynamic light scattering (DLS) will be used to visualize the structural properties of our dispersions. We believe that our suggested combination of experiments and modeling will guide us toward a better understanding of interactions and temperature dependence in polymer/particle dispersions.

## Experimental Section

### Materials and Methods

PS core–shell particles, grafted with PEG, were prepared according to the protocol described in our previous work.^18^ Using a dispersion polymerization method, styrene monomers in the presence of an initiator and PEG, with a molecular weight of 2 kDa, propagate to form PS particles, which then become surface grafted with short PEG. The presence of 2 kDa PEG provides stabilization to the dispersion at room temperature through steric repulsive forces between the grafted layers. In other words, the grafted PEG chains act as a stabilizing agent. Subsequent dialysis was performed to purify the dispersions.

PEG, with an average molecular weight of 100 kDa, was supplied by Sigma-Aldrich. Deionized Milli-Q water was used for all of the synthesis protocols.

### Dynamic Light Scattering

DLS measurements were made using a Malvern Zetasizer equipped with a HeNe laser, with a wavelength of λ = 633 nm, and a detector positioned at a scattering angle of 173°. A source of light shines through a dilute sample of particles. The intensity and fluctuation of the scattered light are measured and used to study the dynamic properties of the colloidal dispersions. The intensity of the scattered light, *I*, as a function of the wave vector, *q*, as well as the lag time, τ, can be measured. A useful dynamic quantity is the autocorrelation function, *g*_2_, defined as
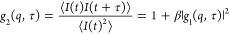
1where β is an instrument constant and *q* is given by
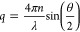
2Here, λ is the wavelength of the incoming light, θ is the angle at which the light scatters, and *n* is the refractive index of the solvent. For a monodisperse system, the function *g*_1_ can be defined as^30,31^
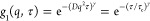
3where τ_c_ is the characteristic relaxation time and γ is the Kohlrausch exponent. Note that  and γ = 1 for diffusive movements. *D* is the Stokes–Einstein diffusion constant. The hydrodynamic radius, *R*_h_, can be then calculated as
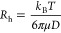
4where μ is the viscosity of the solvent (water).

### Confocal Laser Scanning Microscopy

CLSM was used for imaging and analysis of cluster formations in the samples. CLSM snapshots were monitored on a Leica TCS SP5 scanning microscope, operated in the inverted mode, and equipped with a HeNe laser, λ = 543 nm. Using two cover glasses, separated by a 120 μm spacer, and a 100×/1.4 NA oil immersion objective, imaging of samples was performed by operating the microscope in a bright-field mode.

## Theoretical Modeling

We recall that the experimental system is composed of PS particles carrying grafted 45-mers (2 kDa PEG) and dissolved 2274-mers (100 kDa PEG). In our earlier work, where these PEG-grafted PS particles were synthesized, the grafting density, γ, was measured as γ = 0.355/nm^2^.

We shall adopt a flat surface geometry to work out free energies per unit area and then the Derjaguin approximation (DA) to establish a PMF between the particles.^32^ The polymer-mediated interactions are short-ranged compared to the particle size, so the DA is expected to be nearly exact for these systems. These conditions also ensure that many-body interactions are negligible; that is, the system can be accurately described by pairwise additive PMFs acting between the colloidal particles. From CLSM images, we estimate an average particle diameter of 2300 Å.

A previously established simulation model for PEG in water at room temperature^33^ utilized a hard-sphere description of the monomers. The optimized diameter was found to be 2.65 Å. Neighboring monomers in a chain were connected by rotationally flexible bonds, but with a fixed bond length of *b* = 4 Å. Since we are aiming for a temperature-dependent model, we have used a hard-sphere plus attractive L-J description of the monomers; that is, all monomers interact via ϕ(*r*), defined as
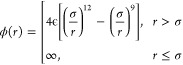
5where *r* is the separation between the monomers. Guided by the low-temperature (good solvent) simulation work, we have set σ = 2.65 Å. Our PMF calculations are based on *classical statistical–mechanical* density functional theory (DFT); that is, they do not involve quantum mechanical calculations. We have utilized the so-called generalized Flory dimer approximation of excluded volume effects.^34,35^ The full polymer DFT formulation is quite lengthy but rather standard by now. We provide a condensed description below. DFT is usually formulated such that nonlocal excluded volume effects are (approximately) accounted for at a monomeric length scale. However, for polymeric molecules, intrachain correlations are not appropriately managed. The influence of other monomers via excluded volume and soft interactions is treated in a “smeared-out” manner, which in our flat surface geometry implies an assumption that the densities only vary with the distance (*z*) normal to the surfaces but is uniform along the lateral directions (*x* and *y*). This tends to be an accurate approach at high monomer densities, such as within the grafted layers. For instance, an earlier work demonstrated excellent agreement between simulated (b* ≡ *b*/σ ≈ 1.5) and calculated monomer density profiles^29^ for fully flexible polymers, provided that the DFT calculations utilized a somewhat increased bond length, b* = 2. This is the bond length we have adopted for all polymers in this work. However, in a dilute solution of isolated chains, the mean-field error is more significant. This is because the monomer density is locally high within “polymer occupied” regions and vanishingly small between these regions. This means that a mean-field approximation of a uniform monomer concentration can be rather poor. As a consequence, the predicted polymer “size” (*R*_*g*_) of our “DFT polymers” is similar to that of ideal chains, which is more compact than they should be. This is because the local monomer density in reality tends to be higher than the corresponding mean-field (“smeared-out”) average. An unfortunate, and well-known, consequence is that the predicted range of polymer-mediated interactions is underestimated. One way to correct for this is to increase the bond length. We have, however, chosen to retain the bond length and instead introduce a bond angle potential between next-nearest neighbors in the ungrafted (dissolved) chains. We thus introduce a potential, *E*_B_, defined as
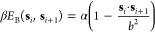
6where β is the inverse thermal energy, whereas **s**_*i*_ denotes the bond vector between monomers *i* and *i* + 1 (i.e., **s**_*i*_ = **r**_*i*+1_ – **r**_*i*_), and α is the strength of the bending potential. Thus, as α increases, the polymers will tend to “stretch out”. The value of α was adjusted so that the low density mean-field prediction matches the one obtained from simulations in the good solvent limit for our target 2274-mers. Specifically, we simulated an isolated 2274-mer using the previously validated model in the good solvent limit^33^ (hard-sphere monomers of diameter 2.65 Å connected by orientationally flexible bonds with a length of 4 Å) and calculated the average degree of gyration, *R*_g_ = ⟨*R*_g_^2^⟩^1/2^. Then we simulated an isolated 2274-mer with point-like (ideal) monomers that were connected with bonds of length *b* = 5.3 Å (b* = 2) but where next-nearest neighbors were effectively repelled, as described by eq 6. The value of α was then adjusted until the *R*_g_ from the good solvent simulation was reproduced. This procedure resulted in a stiffness strength of α = 5.

We still need some way to estimate suitable model values for the energy L-J parameter ϵ or, equivalently, suitable reduced temperatures, T* = *kT*/ϵ, corresponding to our three experimentally investigated temperatures: 20, 45, and 75 °C (roughly). We emphasize that one must keep in mind the “inverted” relation; that is, a *high* “real” temperature corresponds to a high fraction of hydrophobic **B** type monomers which attract each other, which our model is captured by a *low* reduced temperature. It turns out that the theta temperature (UCT for infinitely long polymers) is *T*_C_^*^ = *kT*_C_/ϵ = 7.65 for our polymer model, where the subscript “C” stands for critical. Since we, in the experiments, are investigating the regime of 20–75 °C (293–348 K), that is, below the LCST, we must in our model focus on reduced temperatures *above* 7.65. One way to establish model temperatures that “correspond” to experimental ones (20, 45, and 75 °C) is to borrow results from previous efforts, arriving at the approximate temperature dependence of an “effective” Flory–Huggins (FH) parameter, χ_eff_. The parameter is denoted “effective” as it is temperature-dependent, in contrast to the corresponding parameter in standard FH theory, and thus incorporates entropic effects. This type of FH approach is very much in the same spirit as our current modeling of chains composed of L-J monomers, with temperature-dependent interactions. From Figure 11 in the work by Dormidontova,^28^ we can make crude estimates of χ_eff_ for our three target temperatures. In a commonly adopted simplified approach, the FH “energy” of mixing depends on temperature, Δϵ_mix_(*T*), which of course implies that this should be regarded as a free energy parameter. Borrowing concepts from FH theory, we note that χ_eff_ is proportional to Δϵ_mix_(*T*)/*T*. This means that if we assume that our L-J “energy” (free energy) parameters, ϵ(*T*), scale with temperature in the same manner as Δϵ_mix_(*T*), we have
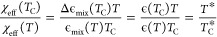
7We thus arrive at a mapping from χ_eff_(*T*) to our reduced temperatures:
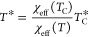
8From standard FH theory, we know that χ_eff_(*T*_C_) = 0.5, and we recall that our mean-field theory predicts *T*_C_^*^ = 7.65. Hence, we arrive at the following mapping between experimental temperatures and reduced temperatures in our L-J model, where the values of χ_eff_(*T*) are estimated from Figure 11 in ref (28):

- 20 °C: χ_eff_(293 *K*) ≈ 0.425 = > T* ≈ 9.0
- 45 °C: χ_eff_(318 *K*) ≈ 0.46 = > T* ≈ 8.3
- 75 °C: χ_eff_(348 *K*) ≈ 0.49 = > T* ≈ 7.8

Extensions of FH theory have been proposed where the interaction parameter varies with composition, but these will not be considered in this work. In order to complete our model, we need to address the interaction between a PEG monomer and the bare PS surface. We shall denote this interaction by *v*_PS_(*z*) and assume that it only varies with the separation, *z*, between the monomers and the PS surface, which is flat in our model. From our previous studies,^18,29^ including our **A**/**B** model, we anticipate a short-ranged hydrophobic attraction between the PEG monomers and the bare PS surface. We also expect this attraction to grow stronger as we increase temperature (in the experimental system), as the fraction of **B** monomers increases. We have chosen to model this attraction by a short-ranged truncated and shifted harmonic potential, acting on all monomers, with a minimum at the (infinitely hard) PS surface:
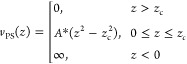
9where *z*_c_ is set equal to one bond length, *b*, ensuring a short-ranged interaction. *A* thus regulates the strength of the attraction. Note that the relevant strength at a given temperature is measured as *A*/(*kT*), that is, how strong the attraction is relative to thermal energy. Thus, with a fixed value of *A*, we will in practice obtain a stronger attraction at “75 °C” (T* = 7.8) than at “20 °C” (T* = 9.0), as *A* is larger compared to *kT* in the former case (7.8 < 9.0). This makes sense given our underlying PEG model, where we anticipate a higher fraction of hydrophobic monomers at higher temperatures.

Our model slit geometry is confined by two “PS surfaces”, located at *z* = 0 and *z* = *h*. The total potential, *V*_PS_(*z*; *h*) from these surfaces is found by superposition: *V*_PS_(*z*; *h*) = *v*_PS_(*z*) + *v*_PS_(*h* – *z*).

Armed with these assumptions and definitions, we need a way to estimate the amplitude *A*. Fortunately, our experimental observations, in combination with DFT predictions, allows us to arrive at a rather specific value of this quantity. As we shall see, this will be further corroborated by agreements between independent predictions and experimental results for a range of different conditions. We will, however, postpone the estimation of *A* until the experimental results have been reported (below).

### Polymer Density Functional Theory

Here, we give a brief account of the polymer DFT that was used to calculate interaction free energies. We refer to earlier work for details.^35−39^ Note that there are two different kinds of polymers in our system: the end-grafted chains, which are short with orientationally flexible bonds, and the dissolved, long, semiflexible polymers.

We let **R** = {**r**_1_,.....,**r**_*M*_} denote the monomer coordinates of a “free” (dissolved) *M*-mer chain (in our case, *M* = 2274). Furthermore, we will by *N*(**R**) denote a multipoint polymer density, from which the densities of separate monomer sites, *n*_*i*_, can be evaluated:
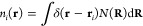
10where δ(**r**) is the Dirac delta function. We also introduce an analogous notation for the grafted chains, which are identified by an index “*g*”.

We note that our model comprises a polymer fluid composed of semiflexible dissolved chains (recall eq 6) in chemical equilibrium with a bulk solution of chemical potential, μ, as well as surface-grafted chains (*M*_g_-mers). The chemical potential is calculated as a derivative of the bulk free energy at the chosen bulk polymer density. These polymers are also under the influence of an external field, *V*_PS_, that originates from our planar PS surfaces at *z* = 0 and *z* = *h*. The grand potential, Ω, can be written as
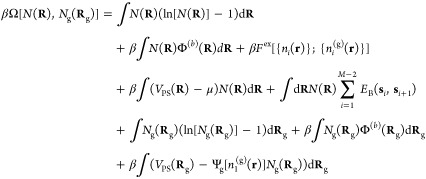
11Here, we note that the mean-field excess free energy, *F*^ex^[{*n*_*i*_(**r**)}{*n*_*i*_^(g)^(**r**)}], originating from *all* monomer–monomer interactions, can be expressed in terms of the site densities, *n*_*i*_(**r**) (dissolved chains) and *n*_*i*_^(g)^(**r**) (surface-tethered chains). These interactions are generally decomposed into separate excluded volume (hard core) and long-ranged attractive parts. In this work, we have managed the former by utilizing the generalized Flory dimer approximation,^34,35^ while the latter is expressed by the attractive part of the L-J interaction. Neighboring monomers along the chain are connected by a bonding potential, Φ^(b)^(**R**), that is defined so that the bond distance is fixed at a constant value *b*:
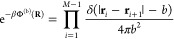
12with an analogous definition for Φ^(b)^(**R**_g_) (where the number of bonds is limited to *M*_g_ – 1 = 44).

In our planar slit geometry, things are simplified by the fact that we can integrate out, in a mean-field manner, all dependencies upon *x*, *y*, leaving only *z*-dependent quantities. We also note that, for our system, the external potential originates from the PS surfaces, modeled as planar walls, with which the monomers interact; see eq 9. Ψ_g_[*n*_1_^(g)^(**r**)] is a Lagrange multiplier, acting on the grafted monomer, which we define as “monomer 1”. Ψ_g_[*n*_1_^(g)^(**r**)] is adjusted so that the desired grafting density is achieved. Monomer 1 is in all grafted chains bonded to one of the surfaces but is allowed to move within one bond length. We formulate these conditions as
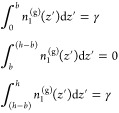
13In passing, we note that Ψ_g_ resembles the Donnan potential in ionic solutions, which is regulated in a similar manner to achieve electroneutrality.^39^

The equilibrium monomer distribution is found by minimizing the grand potential using iterative procedures until self-consistency is obtained.^38,39^ This may seem like a formidable task, but very efficient computational approaches have been developed^38,39^ that are based on recursive relations. Calculations at a given separation and for a given choice of μ (and thus bulk density) are initiated from an estimate of the equilibrium density profiles for monomers belonging to free and grafted chains. An initial educated guess of Ψ_g_[*n*_1_^(g)^(**r**)] is also required. The grand potential minimization leads to integral equations that in turn generates new predictions for the monomer density profiles, as well as Ψ_g_[*n*_1_^(g)^(**r**)]. These integral equations are solved recursively so that all possible conformations of dissolved and grafted chains are accounted for, subject to a *z*-dependent Boltzmann weight. This weight has a mean-field character, although excluded volume correlations, with a range of about a monomer diameter, are approximately taken into account. The bond angle potential, *E*_B_, also introduces correlations between next-nearest neighbors along the chain. In principle, these newly predicted profiles could serve as new guesses for the next iteration. However, such an approach will in most cases lead to divergence, and a more conservative scheme is recommended where only a small fraction of the newly predicted profiles are mixed with the previous profile. This procedure is sometimes referred to as Picard iterations. The iterations continue until self-consistency is achieved.

By calculating the minimized grand potential per unit area, Ω_eq_/*S*, for a range of different separations, *h*, we arrive at a complete free energy curve, which is directly related to the force, *F*, between two identical particles of radius *R*_p_ via the DA:^32^
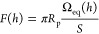
14A cubic spline fit to our discrete set of *F*(*h*) points, followed by yet another integration, generates the final particle–particle PMF, *W*:
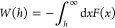
15

A useful and very sensitive test of the written DFT code is that the calculated analytical expression for the normal pressure, −∂Ω_eq_/*∂h*, agrees with its discrete correspondence, −ΔΩ_eq_/Δ*h*. Such tests are not provided here but are obviously available upon request.

## Experimental Results

Particle characterization and thermal analysis of dispersions were carried out using DLS and CLSM analysis. We summarize the results in the following sections.

### Dynamic Light Scattering

According to our DLS measurements, the zeta-potential of the PEGylated PS particles in the absence of any added free PEG was about −3.6 mV, and the ionic strength was also quite low at about 0.06 mM. The DLS data furthermore implied an average particle diameter of 1900 Å, which is slightly smaller than the value obtained from CLSM analyses: 2300 Å.

**Figure 1:**
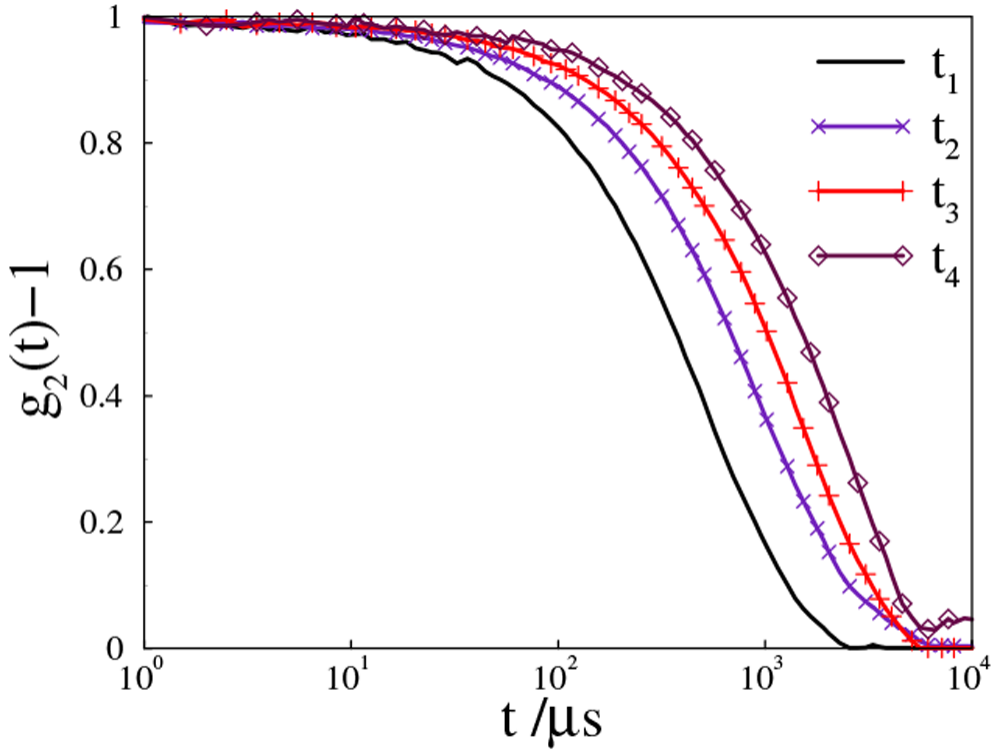
Evolution of aggregates at room temperature in a dispersion of 0.05 wt % particles containing 6 mg/mL PEG with a molecular weight of 100 kDa. Measurements were recorded during a time scale of 2 days with *t*_1_ < *t*_2_ < *t*_3_ < *t*_4_. Here, *t*_1_ indicates the initial state when PEG was added, whereas *t*_2_ = 10 h, *t*_3_ = 24 h, and *t*_4_ = 48 h after polymer addition.

DLS was also employed to analyze the evolution of aggregates in dispersions with added PEG. The autocorrelation function, *g*_2_, of a solution containing particles at a concentration of 0.05 wt %, as well as PEG with a molecular weight of 100 kDa and a concentration of 6 mg/mL, was measured at room temperature. The results are displayed in Figure 1. We note a shift toward slower dynamics as time progresses, which is associated with cluster formation. The diffusion coefficient was also measured, adopting eq 4, and it was found to vary from an initial value of 1.45 to 0.42 μ^2^/s after 48 h. This clearly indicates a transition from fast singular particle movement to slow movements of clusters. Fits to eq 1 indicate diffusive movement of the particles, even at later stages of aggregation. In other words, dispersions of aggregates displayed fluid-like properties.

In order to shed more light on particle evolution, CLSM was also employed, which will be discussed in more detail below (see also the videos in the Supporting Information). A brief note here, based on our observations, is that initial aggregates tended to be rather linear, with substantial branching being developed at later stages.

### Confocal Laser Scanning Microscopy

#### Room Temperature

CLSM was employed to visualize particle aggregation. To this aim, PEG with a molecular weight of 100 kDa was added to the dispersions at different concentrations. Samples of PEG-grafted PS particles were prepared at concentrations ranging from 0.03 to 0.1 wt %. PEG (100 kDa) was then added, arriving at polymer concentrations in the range of 0.7 to 9 mg/mL. The samples were allowed to equilibrate at various temperatures (20–87 °C) and subsequently imaged by a CLSM. The equilibration time varied depending on both temperature and concentration of added PEG. For instance, samples at low temperatures equilibrated on a time scale of 1 day to 2 weeks, depending on the PEG concentration, whereas at higher temperatures, equilibration was fast and the results were extracted within a short period of 1–3 h. Within the intermediate temperature regime, the samples were equilibrated for 2 weeks. CLSM snapshots are collected in Figure 3 for a particle concentration of 0.05 wt % and polymer concentrations between 3 and 6 mg/mL at various temperatures. The confocal videos, supplied in the SI, may provide a better understanding. According to the CLSM measurements, the particle diameter was about 2300 Å, that is, slightly larger than the value obtained from the DLS measurement. Further analysis indicated a particle aggregation at room temperature for a sufficiently high dosage of dissolved polymer. We could detect some degree of clustering at polymer concentrations above 2 mg/mL.

At room temperature, the dissolved polymers generate attractive depletion forces. There are at least two strong arguments in favor of this conclusion. First, we note that if there was any significant attraction between PEG monomers at room temperature, one would expect gel formation at high particle volume fraction, even in the absence of added polymer. However, these dispersions will only form gel at higher temperatures.^19^ Second, if the attraction was caused by bridging at room temperature, then it would become monotonically *stronger* with temperature, as the PEG monomers become progressively more hydrophobic. However, as we shall demonstrate below, there is an intermediate temperature regime (above ambient) where redispersion to a homogeneous sample takes place, clearly suggesting *less* attractive interactions.

Note that a simplifying aspect of these systems is that the presence of a grafted PEG layer will allow us to neglect Hamaker interactions between the PS particles.^40^

**Figure 2:**
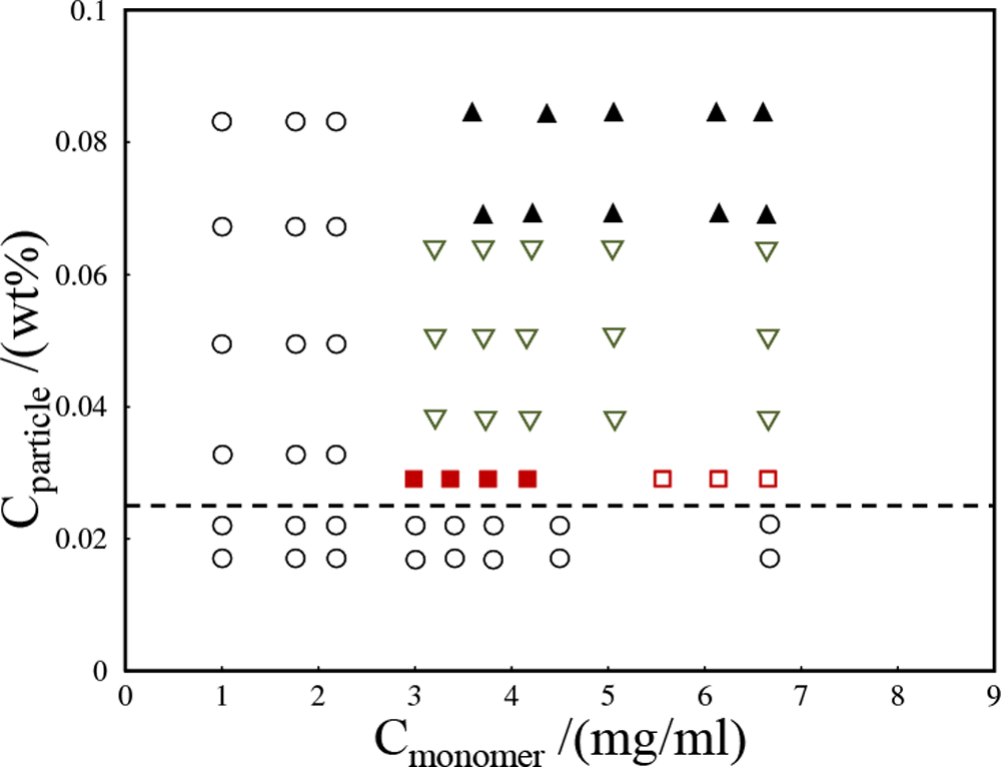
Structural behaviors of particles at room temperature, for varying concentrations of polymer and particle. The molecular weight of free PEG in the experiments was 100 kDa. ○: homogeneous dispersion, stable. ▽: cluster forming metastable dispersions, where phase separation eventually occurs. □: branched clusters that are seemingly stable. ■: stable linear clusters. ▲: two-phase dispersions, i.e., phase separation is rapid.

Our findings from CLSM studies, combined with diffusion analyses, were collected at various particle and polymer concentrations, and the results are summarized in Figure 2. The temperature was kept constant at 20 °C. At rather low polymer concentrations, mixtures of dispersed particles and short strings were observed, and we could in these cases not detect any structural changes with time; that is, the dispersions seemed to reach a stable state. At high concentrations, however, cluster formation quickly slowed down the diffusion of particles, and the dispersions finally displayed demixing, forming two separate phases: a sedimented solid phase and dilute particle dispersion. As is evident from the diagram, the effects of particle volume fraction and polymer concentration on structural properties were rather pronounced. In other words, the presence of a sufficient amount of nonadsorbing dissolved PEG will lead to the formation of clusters and, in some cases, to a complete phase separation.

We briefly mention that we also made some tests where the added PEG chains were quite short, with molecular weights of 2 and 8 kDa. A 40–50 times higher *monomer* concentration was then required to induce depletion flocculation at room temperature. For 2 kDa chains, this corresponds to polymer concentrations that are several thousand times higher than when 100 kDa PEG was used. For these short polymers, we did not detect any obvious structural features of clusters formed, as macroscopic phase separation and further sedimentation were quite rapid.

#### Elevated Temperatures with Added Polymer

**Figure 3:**
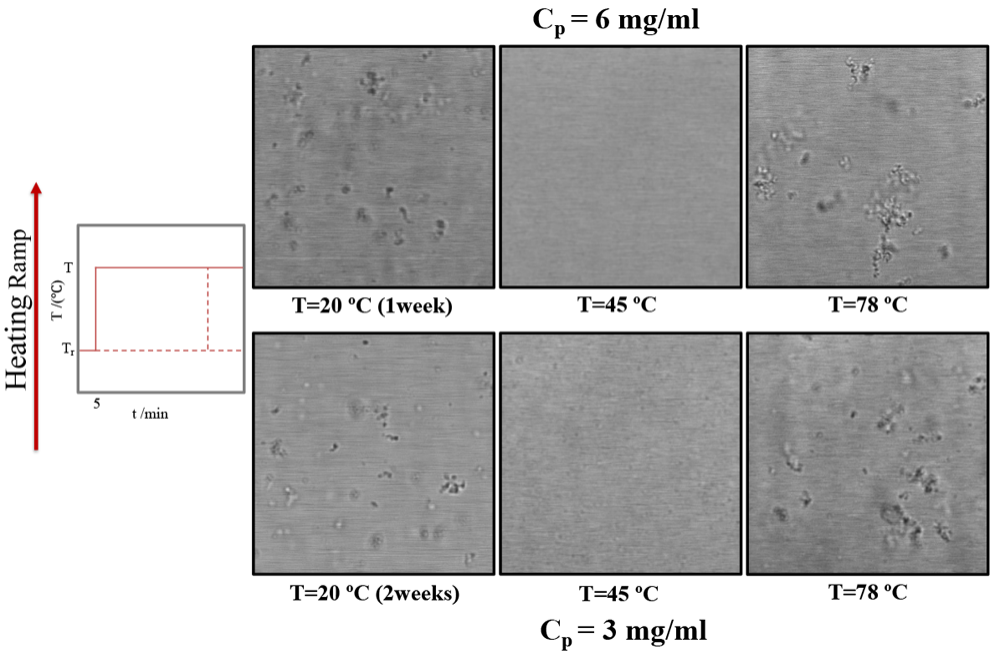
CLSM snapshots of particles at two different concentrations of 100 kDa PEG: 3 and 6 mg/mL. The thermal analysis was performed by heating the samples from *T*_r_ = 20 °C to a temperature *T* ranging from 35 to 78 °C. The temperature sequence was plotted on the left side, indicating two different approaches. In one case, samples were equilibrated at *T*_r_ and *T* directly after preparation. In the other approach, samples that were previously aggregated at *T*_r_ were subsequently heated to *T* and then equilibrated at this temperature. In the latter case, we observed redispersion, i.e., a homogeneous sample, when *T* was about 45 °C.

The measurements that we have reported so far were recorded at room temperature. However, in order to evaluate our theoretical predictions, we performed a thermal analysis on the dispersions at temperatures ranging from *T*_r_ = 20 to *T* = 50, 73, and 78 °C, as illustrated by a heating ramp in Figure 3. This means that particles were either kept at *T*_r_ immediately after polymer addition until they reach equilibrium or were heated to higher *T* immediately after PEG addition and then equilibrated before imaging. An additional experiment was performed on particles which aggregated at low *T*_r_ and were heated to a target temperature, *T*, after aggregation. As shown in Figure 3, we could identify an intermediate temperature regime, around 40–50 °C, where the samples showed no sign of aggregation. A similar result was observed for dispersions which did flocculate at room temperature; that is, they redispersed to form a homogeneous sample as temperature was increased to this interval. Note that this clearly demonstrates that the flocculation observed at room temperature was due to depletion and not bridging attraction. The latter scenario would result in even stronger flocculation at this higher temperature, as a result of the monomers becoming more hydrophobic.

A further temperature increase to about 70–80 °C leads to a pronounced formation of branched clusters.

#### Elevated Temperatures without Added Polymer

**Figure 4:**
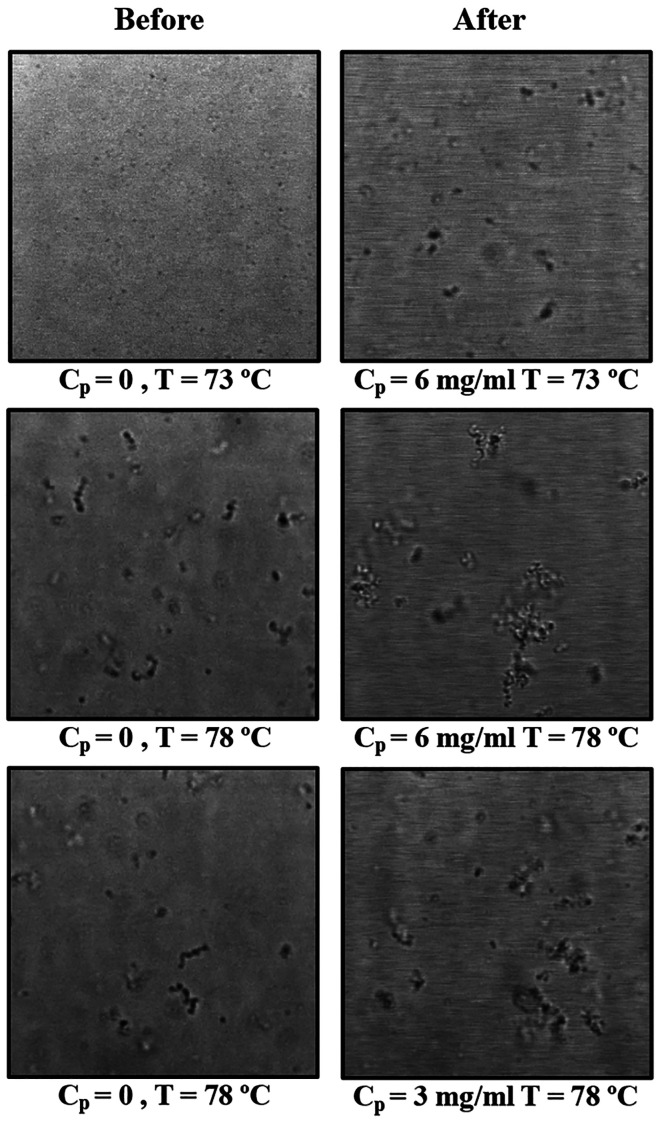
CLSM snapshots of particles before and after PEG 100 kDa addition. On the left panel, no polymer was added. The temperature was kept constant, and images were taken by monitoring the clusters at different time intervals, tracing the evolution toward equilibrium. Samples were allowed to remain at the given temperature for several weeks. The polymer concentration on the right panel was kept at 3 or 6 mg/mL, as indicated below the graphs. At these high temperatures, the aggregates are formed quickly after 1–3 h.

We briefly report CLSM analyses on structures of dispersions that *only* contain PEG-grafted PS particles. We expect that clusters might form in such systems at high enough temperatures, as the PEG layers then become hydrophobic. On the other hand, this is merely a qualitative argument, and it is clearly of interest to establish quantitative results which we subsequently can compare (see below) with predictions from our theoretical model. We have summarized our analyses for the systems in Figure 4. We note that a somewhat higher temperature (by about 5 °C) is required to generate clusters when no polymer is present. Moreover, the presence of dissolved PEG tends to generate more branched clusters, having a more spherical overall shape, than corresponding clusters in the pure particle dispersions.

### Theoretical Modeling

Experimentally, we observe a redispersion of clusters and flocculated structures at roughly 45 °C. This may seem surprising, given the well-established mechanisms according to which equilibrium interactions mediated by dissolved polymers are attractive between nonadsorbing surfaces, due to depletion, but also attractive between adsorbing surfaces via bridging interactions. However, we reiterate that recent work^29^ has demonstrated that in the *intermediate regime*, that is, for intermediately adsorbing surfaces, the polymer-mediated interaction is *repulsive* at equilibrium. These predictions were admittedly based on approximate DFT, but for the special case of ideal chains composed of bonded point-like monomers, DFT results are *exact*, and the predictions were shown to be valid also for those model systems. We provide further analyses of such systems in the Appendix. In summary, these considerations suggest that we can fine-tune the amplitude factor *A*, so that it leads to a net *repulsive* interaction (intermediate adsorption) at T* = 8.3, which we recall is our model representation of the 45 °C experimental system.

**Figure 5:**
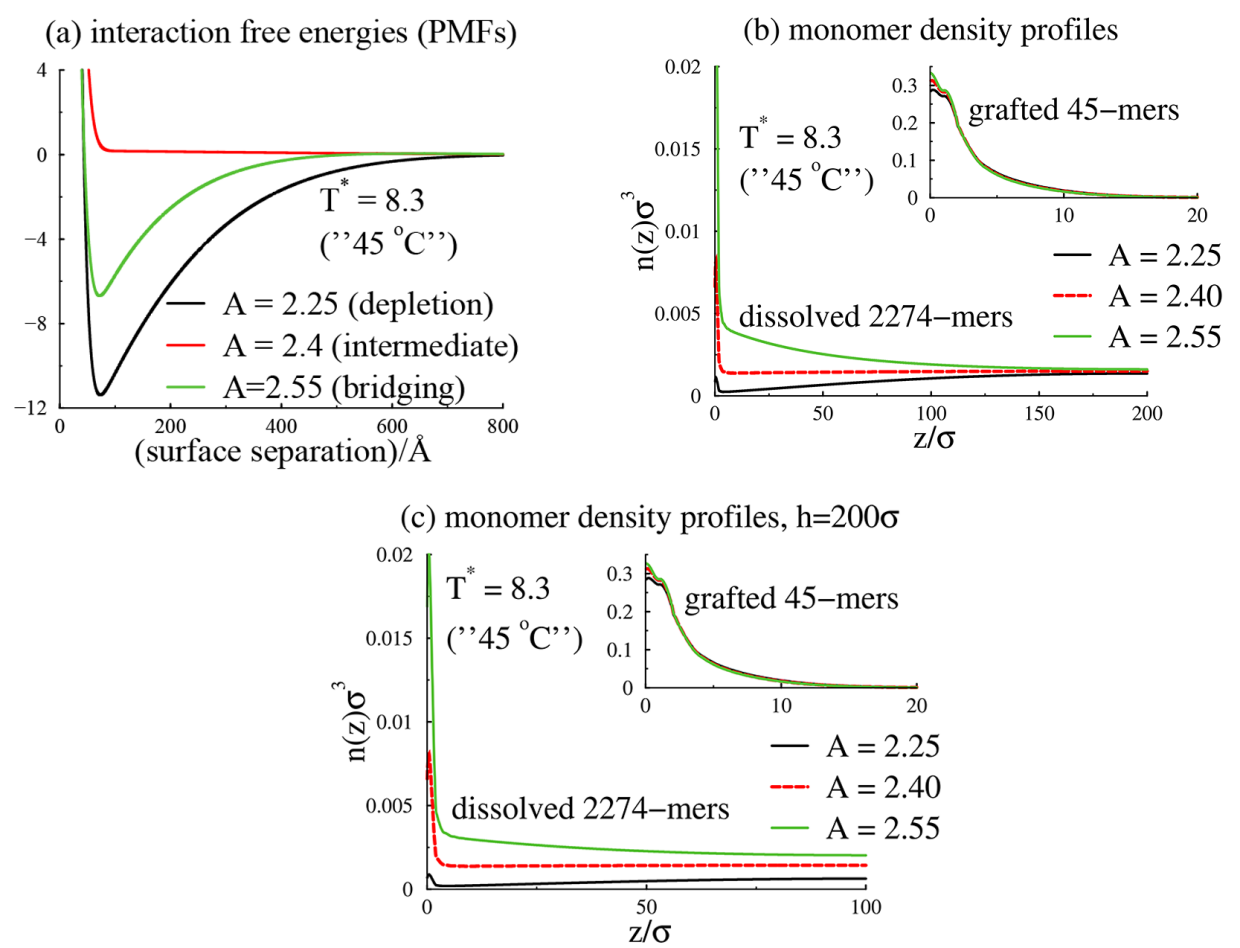
Results from variations of the surface amplitude factor, *A*. (a) Particle–particle pair PMFs, *W*. (b) Monomer density profiles, *n*(*z*), at a single surface. The main graph displays the density profile for monomers belonging to the dissolved 2274-mers, which in turn are in chemical equilibrium with a large (infinite) bulk solution, in which the polymer concentration is 6 g/L, assuming a molecular weight of 100 kDa. The inset shows the density profiles for monomers belonging to grafted chains. The units on the axes of the inset are the same as in the main graph. (c) Monomer density profiles, *n*(*z*), at a separation *h* = 200σ. Otherwise, conditions and notations are the same as in graph (b).

Now we proceed with our *A*-scan, searching for a value that leads to a polymer-induced repulsion at T* = 8.3 (“45 °C”). In Figure 5a, we see that the PMF response to rather modest variations of *A* can be quite dramatic! Across the parameter regime, *A* = 2.25 → 2.55, the model PS surface effectively changes from being nonadsorbing to adsorbing. This is illustrated in graph (b) of Figure 5, where density profiles at a single surface are displayed. For completeness, we also include a graph, (c), where these profiles are given at a shorter separation, *h* = 200σ. In agreement with results in ref (29), the polymer-mediated interaction then swaps from depletion attraction to bridging attractions via an intermediate regime, where the net interaction is *repulsive*. Guided by these results and from experiments, we now fix *A* to a 2.4, whereby the interaction is repulsive for our corresponding system (*T* = 8.3). In other words, we use the observation of redispersion at our intermediate temperature as a way to measure the “proper” strength of the hydrophobic attraction. With *A* = 2.4, the maximum strength of the surface potential, *v*_PS_(*z* = 0), is, at T* = 8.3, slightly larger than thermal energy: β*v*_PS_(*z* = 0) ≈ −1.16.

### Predictions

Now that we have set all model parameters, based on previous works on PEG+water solutions, and our observations of redispersion of our PEG-grafted PS particles at 45 °C, we are ready to make theoretical predictions for PMFs and concomitant structures at higher and lower temperatures (with 45 °C as our reference).

**Figure 6:**
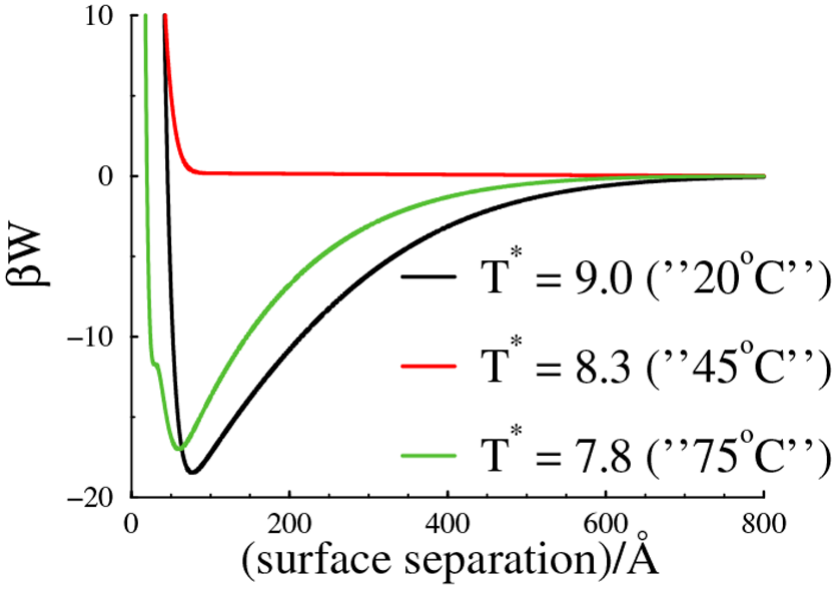
DFT predictions of particle–particle PMFs at three different temperatures. The bulk concentration of the dissolved 2274-mers is 6 mg/mL, assuming a molecular weight of 100 kDa.

Calculated polymer-mediated PMFs at our model representation of 20, 45, and 75 °C are collected in Figure 6. The temperature response is quite remarkable given that the relative difference in absolute temperature (293–348 K) is rather modest. This result, which clearly captures the experimentally observed non-monotonic stability of the dispersion, is the main result of the theoretical modeling part of this work. We note a peculiar “kink” on the PMF curve at narrow separations for the 75 °C (T* = 7.8) system. As we shall see below, this originates from interactions between the grafted layers, which displays an attractive regime at this and higher (real) temperatures.

#### Predictions for Grafted PS Particles without Added Polymers

Using our established model, we can of course also predict PMFs between our grafted particles in the absence of any added polymer. The polymer-induced interactions will then only be due to the tethered chains. At low temperatures, this will lead to a monotonic repulsion, as the entropic cost of reducing the number of available configurations as another surface approach will dominate the weak L-J attractions between the monomers. However, at high temperatures, the latter will provide an attractive regime.

**Figure 7:**
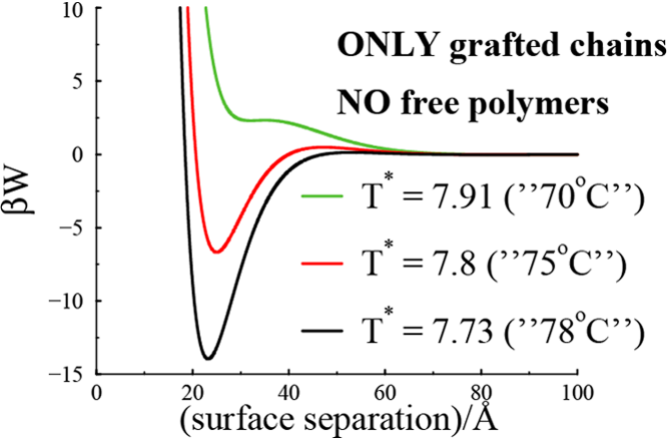
DFT predictions of particle–particle PMFs in the *absence* of any added polymers; i.e., the interactions are mediated by the grafted chains. Three different model temperatures, all of which are close to our T* = 7.8 reference (i.e., “75 °C”), are shown.

According to experiments, clusters will form in samples free of added polymers, at 78 °C, but this is indeed close to the “transition point”; that is, they will not form at temperatures substantially lower than this threshold values. It is obviously of interest to see what our theoretical model predicts at similar temperatures. We have already established a temperature mapping according to which 75 °C “corresponds to” a reduced temperature of T* = 7.8 in our model L-J system. Hence, if we wish to study a temperature regime very close to this value, say, 70–78 °C, it makes sense to utilize a simple linear scaling around this value so that 70 °C corresponds to a model reduced temperature of T* = 7.8 × 348 K/343 K ≈ 7.91, while T* = 7.8 × 351 K/348 K ≈ 7.73 provides a model representation of 78 °C. Calculated PMFs at these temperatures are shown in Figure 7. It is quite clear that our model predicts a transition very close to 75 °C, in perfect agreement with experimental findings, without any adjustments of the already established theoretical model! We have to admit that this kind of quantitative agreement probably is, to some extent, fortuitous. It is clear that the “kink” of the T* = 7.8 curve in Figure 6 originates from attractions between grafted polymers. Note the different separation scale between Figures 6 and 7. The direct attraction between grafted layers has, as expected, a much shorter range than that mediated by the long dissolved chains.

There is another aspect worth noting. According to our CLSM analyses, the clusters that are formed at about 78 °C in the absence of added PEG tend to be more linear than when the dissolved polymers mediate the attraction. This is in agreement with the prediction above of a substantially more short-ranged attraction in samples free of dissolved polymers. Note that we have not included an expected weak but long-ranged electrostatic repulsions, given that DLS data suggest a weak particle charge and a low ionic strength. Recent simulation work^41^ has demonstrated that, in the presence of such a double-layer barrier, the width of the adhesive minimum has a strong impact on the structure of the clusters. Specifically, a narrow attraction leads to more linear clusters, which is exactly what we observe in this work.

## Conclusions

Based on previous theoretical modeling,^29^ we predicted that our experimental system would display a non-monotonic temperature dependence where flocculation at high and low temperatures (for a sufficiently high dosage of added polymer) is replaced by a fully homogeneous dispersion at some intermediate temperature. This qualitative prediction was indeed confirmed by our experimental investigations. Using the experimental data, we have constructed a designated (specific) coarse-grained model for this particular system, whereby semiquantitative agreements can be achieved using a minimum number of parameters. We emphasize that all our measurements and calculations were performed at supracritical conditions, which experimentally corresponds to temperatures considerably below the bulk LCST. Hence, we do not expect any polymer configurational transitions, such as a coil to globule transition.

We hope that our work may pave the way for new options to regulate colloidal stability, where polymer addition is combined with changes of solvent quality using (for instance) pH, salt, or temperature control.

## Figures and Tables

**Figure 8:**
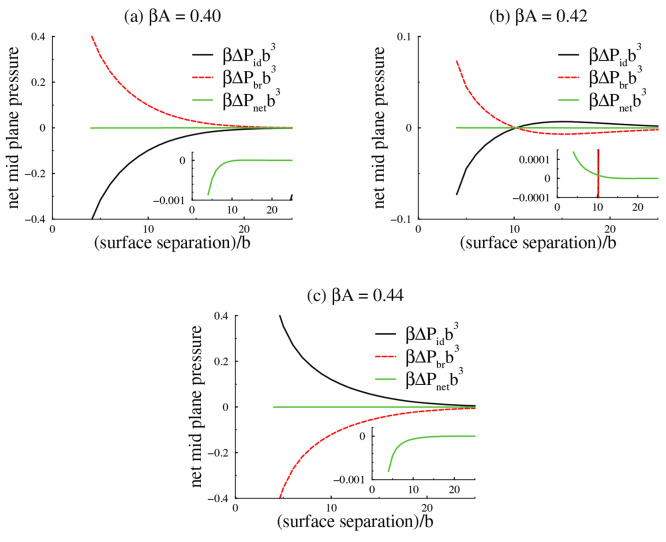
Net pressure contributions across the midplane for surfaces with various affinity (*A*) immersed a solution containing dissolved ideal 100-mers (*n*_b_*b*^3^ = 1). The inset in each graph displays a zoom-in, focusing on the net pressure (same units on the axes as in the main graph). Note that Δ*P*_net_ = Δ*P*_br_ + Δ*P*_id_. (a) β*A* = 0.40; (b) β*A* = 0.42. Notice the difference in scale, compared to (a) and (c). (c) β*A* = 0.44.

## Appendix: Using an Ideal Chain Model to Elucidate the Origin of the Repulsion

The DFT treatment is *exact* for ideal polymers composed of point-like monomers that do not interact with each other—a commonly used model for theta solvent conditions. Another advantage with this model is that the results are easier to interpret and scrutinize. We shall therefore devote this Appendix to analyses of surface interactions in the presence of ideal nongrafted 100-mers, with orientationally flexible bonds (no *E*_B_ potential). These will generate a familiar behavior as the surface affinity is increased with a polymer-mediated repulsion at intermediate adsorption strengths and attractions at significantly higher (bridging) or lower (depletion) affinities. It is instructive to analyze separate net pressure contributions across the mid plane.^42^ We recall that the range of the surface potential is only one bond length, *b*, from either surface. Hence for separations exceeding 2*b*, there are only two contributions to the polymer-mediated pressure across the mid plane, namely, the overall repulsive ideal entropic pressure, *P*_id_, and the attractive bridging pressure, *P*_br_. The former is given by the monomer density at the mid plane, i.e., β*P*_id_ = *n*(*h*/2), whereas we define the latter as the number of bonds crossing the midplane at a perpendicular orientation.† These two contributions have a tendency to be large and almost cancel out. For instance, in the bulk solution, β*P*_id_^(b)^ = *n*_b_, where *n*_b_ is the bulk monomer density. The total osmotic pressure is given by the bulk polymer concentration, i.e., β*P*^(b)^ = *n*_b_/*M* (recall that *M* is the degree of polymerization). In other words, the bulk bridging pressure is *βP*_br_^(b)^ = *n*_*b*_(1/*M* – 1). For a 100-mer polymer solution, this means that the separate ideal and bridging pressures are about 100 times stronger than the net pressure.

More relevant to the net pressure is how these contributions vary relative to their corresponding values in the bulk, i.e., Δ*P*_id_ ≡ *P*_id_ – *P*_id_(*h* → *∞*) and Δ*P*_br_ ≡ *P*_id_ – *P*_br_(*h* → *∞*).These quantities are plotted across the transition regime from depletion to bridging in Figure 8. As expected, the net bridging pressure, Δ*P*_br_, is negative for a strong surface potential and positive in the depletion regime. The net ideal pressure will display an opposite trend. At intermediate conditions, the outcome is less obvious. It turns out that the ideal pressure, which we recall is slightly stronger than the bridging pressure in the bulk, displays a somewhat more dramatic variation with *A* than the bridging pressure. This leads to a surface affinity regime where the net bridging pressure is more repulsive than the ideal pressure is attractive.

In a model where monomer–monomer interactions are included, these analyses become more complicated,^42^ but the main driving force is the same; that is, the repulsion arises as a consequence of a difference between how strongly the various pressure contributions respond to a variation of the surface affinity in the crucial transition regime where these net contributions change sign.

## Supporting Information Available

Further details of supporting video files (PDF)
Confocal scanning recordings of dispersions: 3 mg/mL at 78 °C (Video S1, AVI); 3 mg/mL at room temperature (Video S2, AVI); 6 mg/mL at 73 °C (Video S3, AVI); 6 mg/mL at 78 °C (Video S4, AVI); intermediate (Video S5, AVI); no PEG at 73 °C (Video S6, AVI); no PEG at 78 °C (Video S7, AVI); 6 mg/mL at room temperature (Video S8, AVI)

## Supplementary Material

ng1c00011_si_001.pdf

ng1c00011_si_002.avi

ng1c00011_si_003.avi

ng1c00011_si_004.avi

ng1c00011_si_005.avi

ng1c00011_si_006.avi

ng1c00011_si_007.avi

ng1c00011_si_008.avi

ng1c00011_si_009.avi
