# Supplementary material for: From Attraction to Repulsion to Attraction: Non-monotonic Temperature Dependence of Polymer-Mediated Interactions in Colloidal Dispersions
Source: ACS Nanosci Au. 2021 Aug 25;1(1):69–80. doi: 10.1021/acsnanoscienceau.1c00011 (PMC10125165; doi:10.1021/acsnanoscienceau.1c00011)
Supplement: Supplementary file 1 — ng1c00011_si_001.pdf [file ng1c00011_si_001.pdf]

From Attraction to Repulsion to Attraction: Non-monotonic Temperature Dependence of Polymer-Mediated Interactions in Colloidal Dispersions.

Sara Haddadi, Marie Skepö and Jan Forsman*

Theoretical Chemistry, Lund University, P.O.Box 124, S-221 00 Lund, Sweden

E-mail: jan.forsman@teokem.lu.se,phone:int+46462220381

Supplementary Videos

All the videos were recorded by confocal scanning microscopy, whereby it is possible to identify particle cluster formation (or redispersion), as a response to a temperature variation, and polymer addition. The particle concentration was kept constant 0.05 wt%, for all displayed videos. The concentration of the added PEG, as well as the temperature, were then adjusted. Bright field mode was chosen for recording the videos, at a frame rate of 15 fps. There are three sets of videos, for which we provide short explanations below.

Samples without added PEG

These videos were recorded in the absence of any PEG, at 73 °C (Video S6), and 78 °C (Video S7). The polystyrene (PS) particles, grafted with short PEG (2 kDa) started to aggregate at 78 °C, mainly aligning linearly at such conditions. Below this threshold temperature, particles seemed to remain well dispersed.

Intermediate temperature regime

This video, denoted **Video S5**, shows a stable dispersion of particles, at an inter-mediate temperature, 45 °C, in the presence of added PEG (100 kDa). There is here no tendency for the particles to aggregate. Such a redispersion was also observed for all the samples with a PEG concentration below 10 mg/ml, within a temperature interval of about 35 to 45 °C. We only include one video to illustrate these conditions.

High and low temperatures, with added PEG

Videos with names **Videos S1-S4 and S8** were recorded at conditions where PEG (100 kDa) was added and were the particles aggregated to form clusters. The "C" indicates the concentration of added PEG, whereas "T" is the temperature at which videos were recorded. The PEG concentration was adjusted to 3 and 6 mg/ml and temperature was varied from 20 to 73 and 78 °C. The labels show the values of each concentration and temperature.
